# ORAL montelukast versus inhaled beclomethasone in the prophylactic treatment of post ACUTE viral bronchiolitis wheezing

**DOI:** 10.1186/1939-4551-8-S1-A128

**Published:** 2015-04-08

**Authors:** Luciana Zignago Moreira Dos Santos, Chalene Guimarães Soares, Wilson Rocha Filho

**Affiliations:** 1 Fhemig, Brazil

## Background

Acute viral bronchiolitis is one of the most common respiratory infections in infancy, leading to the hospitalization of about 1-2% of infected infants. A number of studies have suggested that hospitalization due to bronchiolitis could increased the risk of developing pulmonary sequelae, specially recurrent cough and wheezing.

## Methods

This is a randomized, pilot study, involving infants ≤ 1 year of age hospitalized due to acute viral bronchiolitis. All patients with moderate-severe bronchiolitis (Wang score ≥ 8) are eligible to initiate the study. At hospital discharge, recruited patients are randomized into three study groups - conventional treatment, oral montelukast (MK) or inhaled beclomethasone dipropionate (BDP) - and directed to monthly follow-up appointments for 6 months. We aim to determine the potential benefit of inhaled BDP and oral MK in the natural history of post bronchiolitis wheezing. The primary variables are: need of hospitalization and number of visits to the emergency department (ED). The secondary variables are: number of days until first exacerbation, duration of hospitalization, number of asymptomatic days and need of bronchodilator and/or oral corticosteroid.

## Results

Until this moment, 47 patients were randomized, out of which, 26 ended the study, being 9 in the no treatment group(A), 11 in the oral MK group (B) and 6 in the inhaled BDP group (C). Considering the primary variables, only one hospitalization was reported in the no treatment group A. Among group A, 3 visits to the ED were reported and 5 infants needed to use albuterol spray (1-3 times); 1 patient was excluded. Among the 11 patients in the oral MK group B, 2 infants developed a wheezing episode following a viral respiratory infection, 2 visits to the ED were reported and 4 infants needed to use albuterol spray (1-2 times); 2 patients were excluded. Among the 6 patients in the inhaled BDP group C, 2 infants needed to use albuterol spray (1-3 times); 2 patients were excluded.

## Conclusions

To the extent of our knowledge, there are no studies comparing inhaled BDP and oral MK in the prophylaxis of post acute viral bronchiolitis wheezing. A larger number of patients will allow us to establish more significant statistical data. More studies are necessary to establish the presumable benefit of MK and/or inhaled BDP in the history of post bronchiolitis recurrent wheezing.

